# Clinical Outcomes and Treatment Strategies in Catastrophic High-Risk Pulmonary Embolism: A Retrospective Analysis

**DOI:** 10.3390/jcdd12120459

**Published:** 2025-11-25

**Authors:** María Caridad Mata, Ignacio Español, Arantxa Gelabert, Jesús Aibar, Núria Albacar, Elena Sandoval, Pedro Castro, Sònia Jiménez, Jeisson Osorio, Jorge Moisés

**Affiliations:** 1 Department of Respiratory Medicine, Hospital Clínic de Barcelona, Institut d’Investigacions Biomèdiques August Pi i Sunyer (IDIBAPS), University of Barcelona, 08036 Barcelona, Spain; 2 Radiology Department, Centre de Diagnòstic per la Imatge, Hospital Clínic de Barcelona, IDIBAPS, University of Barcelona, 08036 Barcelona, Spain; 3 Deparment of Internal Medicine, Institut Clínic de Medicina i Dermatologia (ICMID), Hospital Clínic de Barcelona, IDIBAPS, University of Barcelona, 08036 Barcelona, Spainpcastro@clinic.cat (P.C.); 4 CIBER of Respiratory Diseases (CIBERES), 28029 Madrid, Spain; 5 Department of Cardiovascular Surgery, Hospital Clínic de Barcelona, IDIBAPS, University of Barcelona, 08036 Barcelona, Spain; 6 Medical Intensive Care Unit, Institut Clínic de Medicina i Dermatologia (ICMID), Hospital Clínic de Barcelona, IDIBAPS, University of Barcelona, 08036 Barcelona, Spain; 7 Emergency Department, Hospital Clínic de Barcelona, IDIBAPS, University of Barcelona, 08036 Barcelona, Spain

**Keywords:** pulmonary embolism, high risk, systemic thrombolysis

## Abstract

High-risk pulmonary embolism (PE) is a life-threatening condition characterized by hemodynamic instability, often leading to catastrophic outcomes such as cardiac arrest and cardiogenic shock. We conducted a retrospective analysis of patients diagnosed with high-risk PE at a single tertiary center between 2018 and 2024. Catastrophic PE was defined as high-risk PE with hemodynamic collapse, including cardiac arrest and/or the requirement for high-dose vasopressors. Data on clinical characteristics, treatments, and outcomes were analyzed. Catastrophic PE accounted for 59% of cases. Systemic thrombolysis was the most frequent reperfusion strategy (67%), while catheter-directed therapies (35.4%) and VA-ECMO (11.4%) were used selectively. Despite aggressive management, catastrophic PE exhibited significantly higher mortality rates at 7 days (40%) and 30 days (49%) compared to non-catastrophic cases (9% and 12.5%, respectively). These patients also showed higher rates of multiorgan failure and required more invasive support. This study underscores the importance of early recognition and tailored treatment strategies for catastrophic PE, highlighting its distinct clinical presentation and worse outcomes compared to non-catastrophic high-risk PE. Further research is essential to refine treatment protocols and improve survival in this critically ill population, emphasizing the utility of a standardized classification to enhance clinical management and research consistency.

## 1. Introduction

High-risk pulmonary embolism (PE) is characterized by the presence of hemodynamic instability or obstructive shock at presentation, conditions strongly linked to high mortality rate. Despite variability among studies, an in-hospital mortality rate of 28% is reported for high-risk PE, increasing to over 65% in cases of cardiocirculatory arrest [1]. These discrepancies likely reflect differences in clinical severity, particularly the presence of profound obstructive shock, rather than hypotension alone. Importantly, high-risk PE encompasses not only low arterial pressure but also broader indicators of hemodynamic compromise, which may necessitate distinct therapeutic approaches.

Catastrophic PE is a term now increasingly recognized to describe the most critical cases of high-risk PE characterized by hemodynamic collapse, (those requiring high-dose vasopressors due to concern for impending cardiac arrest or experiencing cardiac arrest with or without cardiopulmonary resuscitation efforts) [2]. This study provides one of the first European perspectives to adopt the terminology of catastrophic PE, aiming to clarify its clinical significance and explore specific treatment outcomes.

This study offers one of the first European perspectives to adopt the term catastrophic pulmonary embolism (PE), aligning with emerging literature to better characterize the most critically ill subset of high-risk PE patients. By applying this terminology, we aim to clarify its clinical relevance and explore its implications for treatment and outcomes. Specifically, the objective of this study was to compare the clinical characteristics, management strategies, and mortality rates between patients with catastrophic and non-catastrophic high-risk PE treated at a single tertiary center.

## 2. Materials and Methods

We conducted a retrospective analysis of all patients diagnosed with high-risk PE at our single tertiary center from 2018 to 2024. High-risk PE was defined as hypotension with sustained systolic blood pressures below 90 mmHg, a drop of 40 mmHg or more from usual systolic blood pressure, or the need for vasopressor support [3]. At presentation, catastrophic PE was defined per Kobayashi et al.: those requiring high-dose vasopressors due to concern for impending cardiac arrest or experiencing cardiac arrest with or without cardiopulmonary resuscitation efforts [2]. High-dose vasopressors were defined as noradrenaline (NAd) dosing exceeding 0.4 μg/kg/min or the concurrent use of two vasopressors [4]. Non-Catastrophic High-Risk PE included patients with sustained hypotension who did not meet the criteria for catastrophic PE. Haemorrhagic complications were classified as major bleeding episodes according to the Control of Anticoagulation Subcommittee of the ISTH [5], and reperfusion failure was defined as persistent clinical instability within the first 36 h [6]. This study was reported in accordance with the STROBE (Strengthening the Reporting of Observational Studies in Epidemiology) guidelines (Supplementary Materials Table S1).

### Data Collection and Statistical Analysis

Data on clinical characteristics, laboratory results, imaging tests, treatments, and outcomes were collected. Statistical analysis included the Mann–Whitney U test for non-normally distributed continuous variables, and Kaplan–Meier survival analysis. Categorical variables were compared using chi-squared or Fisher’s exact tests. A two-sided *p*-value of less than 0.05 was considered statistically significant.

## 3. Results

During the study period, 79 patients with high-risk PE were included. Most of the patients were female (54.4%), with a mean age of 61.8 years and a body mass index of 29.9 kg/m^2^. The diagnosis of PE was confirmed by computed tomography pulmonary angiogram (CTPA) in 96.2% of patients, and formal transthoracic echocardiography (TTE) was performed in 32.9%. Half of all patients (50.6%) experienced cardiac arrest at presentation, requiring cardiopulmonary resuscitation (CPR) with a median duration to Return of Spontaneous Circulation (ROSC) of 24 min. Most patients required vasopressors (75%) and mechanical ventilation (58.4%). Veno-Arterial Extracorporeal Membrane Oxygenation (VA-ECMO) was performed in 9 patients (11.4%). Systemic thrombolysis (ST) was the most common reperfusion therapy, administered to 67% of patients, followed by catheter-directed therapies (CDT) in 35.4%, some patients received more than one therapy. Isolated anticoagulation was administered to 8 (10.1%) patients, and one patient underwent surgical embolectomy. Combined reperfusion therapies were employed in 19 (24%) patients due to reperfusion failure: CDT was performed in 17 patients following ST, and ST was administered to 2 patients after CDT. Endovascular thrombectomy accounted for the majority of CDT cases (25 out of 28; 89%). The median ICU length of stay (LOS) was 5 days, with a total LOS of 13 days. Major bleeding occurred in 25.3% of patients. Unadjusted 7-day mortality was 27.8%, with 30-day and 90-day all-cause mortality rates of 34.2% and 36.7%, respectively (Appendix A Table A1).

Differences between patients with catastrophic and non-catastrophic high-risk PE are summarized in Table 1. Forty-seven patients were classified as catastrophic high-risk PE (59.5%). Catastrophic PE patients were more likely to present with syncope (61.7% vs. 31.3%; *p* = 0.005), lower mean arterial pressure (65 mmHg vs. 76 mmHg; *p* = 0.012) and higher heart rate (102 bpm vs. 87 bpm; *p* = 0.029). Blood tests revealed significantly higher serum lactate levels (74.9 mg/dl vs. 12.7 mg/dl; *p* ≤ 0.001), SpO2/FiO2 ratio (203 vs. 260; *p* = 0.013), PaCO_2_ (54.2 mmHg vs. 35.6 mmHg; *p* ≤ 0.001), creatinine (1.54 mg/dl vs. 0.94 mg/dl; *p* ≤ 0.001), High-sensitivity Troponin I (1727.7 ng/mL vs. 44.2 ng/mL; *p* = 0.011) and liver enzymes. They also required mechanical ventilation more frequently (81% vs. 22%; *p* ≤ 0.001) and were more likely to need vasopressors. Multiorgan failure was more common in catastrophic PE (34% vs. 6.3%; *p* = 0.002). Consequently, SAPS II and SOFA severity scoring were higher too (76 vs. 30; *p* < 0.001 and 11 vs. 5; *p* < 0.001, respectively).

Regarding treatment, patients with catastrophic PE were more likely to receive ST (89% vs. 43.8%; *p* = 0.001) and VA-ECMO (19%vs 0%; *p* = 0.009) without significant differences in the use of CDT (34% vs. 37.5%; *p* = 0.830). Reperfusion failure was more common in catastrophic PE patients (34% vs. 9.4%; *p* = 0.015).

**Figure 1:**
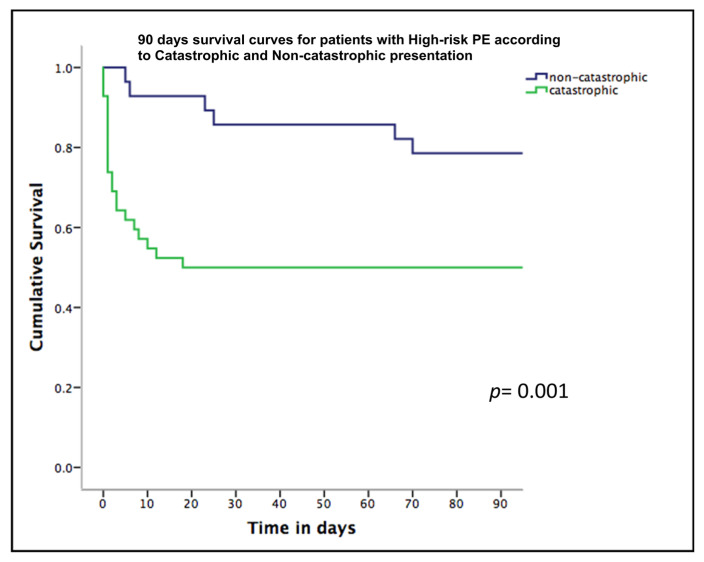
Kaplan–Meier 90-day mortality curves comparing patients diagnosed with catastrophic Pulmonary Embolism and non-catastrophic Pulmonary Embolism.

Finally, unadjusted 7-day, 30-day, and 90-day hospital mortality rates were significantly higher in catastrophic PE patients compared to non-catastrophic patients (7-day: 40.4% vs. 9.4%, *p* = 0.002; 30-day: 49% vs. 12.5%, *p* = 0.002; 90-day: 49% vs. 19%, *p* = 0.024), as illustrated in Figure 1. Catastrophic PE patients had also shorter LOS (7 days vs. 15 days; *p* = 0.05), with no significant differences in major bleeding compared to patients with non-catastrophic high-risk PE.

## 4. Discussion

This study presents a cohort of high-risk PE patients with a 30-day mortality rate of 34% and a major bleeding rate of 25.3%. These results align with previous observational studies, which have reported mortality rate ranging from 20% to 60% [7,8] as well as similar patterns of bleeding complications. Notably, most patients in our cohort received reperfusion therapies, with systemic thrombolysis being the most frequent (65%), contrasting with earlier reports that indicated lower rates of reperfusion in real-world settings [9]. Our findings highlight key differences in clinical presentation, treatment requirements, and outcomes between patients with catastrophic high-risk PE and those with high-risk PE without hemodynamic collapse. Patients with catastrophic PE exhibited more profound hemodynamic instability, higher rates of multiorgan failure, and were more likely to receive mechanical ventilation and VA-ECMO. Importantly, this group also demonstrated markedly elevated serum lactate levels (74.9 mg/dl vs. 12.7 mg/dl) alongside other markers of organ dysfunction such as creatinine, PaCO_2_, liver enzymes, and High-sensitivity Troponin I. The significantly higher lactate levels observed in catastrophic PE patients suggest that lactate may serve as a useful biomarker to identify individuals at risk of progressing to hemodynamic collapse. This opens the possibility of using lactate to further stratify patients within the sustained hypotension phenotype, potentially distinguishing those with early signs of obstructive cardiogenic shock. While previous studies have shown that elevated lactate levels in hemodynamically stable PE patients can predict adverse outcomes [10], its role in high-risk PE populations remains less defined and warrants further investigation.

Additionally, patients with catastrophic PE had a higher rate of systemic thrombolysis (83.3% vs. 46.4%), reflecting the urgency for reperfusion in severely unstable patients, where invasive techniques may be less feasible. No statistically significant differences in bleeding complication rates were observed between the groups. However, no conclusions regarding the effectiveness or safety of thrombolysis can be drawn from these findings, as this represents a particularly severe subgroup of patients. These findings are consistent with Kobayashi et al., who described a similar trend in more critically ill patients [2]. However, our cohort had a higher incidence of cardiac arrest at presentation (50.6%), which may explain why our patients were twice as likely to receive systemic thrombolysis in comparison. Endovascular thrombectomy was less common in this group, whereas in non-catastrophic PE, both strategies were used at similar rates. Surgical intervention was performed rarely, consistent with current practice, where surgery is reserved for cases in which other reperfusion options are contraindicated or unsuccessful. Our findings are further contextualized by the recent work of Stadlbauer et al., who reported on a large cohort of high-risk PE patients, emphasizing a broader management approach [11]. Their observations, particularly regarding the benefit of VA-ECMO for stabilization in severe presentations to facilitate subsequent reperfusion strategies, align with the clinical course and interventions observed in our catastrophic PE cohort. This underscores the evolving paradigm for managing the most critically ill PE patients, where advanced hemodynamic support, such as VA-ECMO, plays a crucial role in enabling definitive therapies. In our cohort, the nine patients who required VA-ECMO had a 0% 30-day mortality rate; only one patient died after 120 days due to withdrawal of support in context of advanced hepatocarcinoma. The low in-hospital mortality rate in our cohort should be interpreted cautiously, considering the potential for selection bias. Importantly, this study is among the first European investigations to use the term catastrophic PE, marking a further step in standardizing terminology for more precise patient stratification and treatment strategies.

## 5. Conclusions

These findings underscore the importance of early identification and tailored management strategies for catastrophic PE. Given the high mortality and complexity of treatment in this very high-risk population, further research is needed to refine treatment protocols and explore additional therapeutic options to improve patient outcomes. Furthermore, this study emphasizes the utility of the term catastrophic PE, paving the way for its broader adoption in clinical practice to enhance stratification and management of the most critically ill patients.

## 6. Limitations

Limitations of the present study are its descriptive and uncontrolled design as well as its single-center setting, which may affect the generalizability of the findings. Additionally, the relatively small sample size, particularly in the subgroup analyses, reduces the statistical power.

## Figures and Tables

**Table 1:** Baseline characteristics in Catastrophic and Non-catastrophic High-risk PE.

	Catastrophic PE n = 47	High-Risk PE n = 32	*p* Value
**Demographics**			
Age [years], ± SD	60.4 ± 16.5	63.8 ± 13.2	0.614
Sex [male], *n* (%)	19 (40.4)	17 (53.1)	0.077
Body mass index [kg/m^2^], ± SD	28.6 ± 6.2	31.7 ± 9	0.495
**Past medical history**			
Smoking, *n* (%)	18 (38.3)	14(47.8)	0.815
Arterial hypertension, *n* (%)	16 (34)	15 (53.12)	0.108
Dyslipidemia, *n* (%)	14 (29.8)	15 (46.9)	0.156
Diabetes mellitus, *n* (%)	6 (12.8)	5 (15.6)	0.750
History of stroke, *n* (%)	2 (4.3)	3 (9.4)	0.475
History of liver disease, *n* (%)	1 (2.1)	2 (6.3)	0.383
History of cancer, *n* (%)	7(14.9)	9 (28.1)	0.167
COPD, *n* (%)	4 (8.5)	5(13.5)	0.473
Atrial fibrillation, *n* (%)	0	3 (9.4)	0.063
Chronic heart failure, *n* (%)	0	1 (3.1)	0.405
Coronary artery disease, *n* (%)	0	2 (6.3)	0.161
Chronic renal disease, *n* (%)	4 (8.5)	2 (6.3)	0.533
**Predisposing factors for venous thrombo-embolism**			
Hormonal contraceptives (%)	4 (8.5)	0	0.140
Surgery 1–2 weeks previous (%)	7 (14.9)	6 (18.8)	0.764
Prolonged bed rest, *n* (%)	10 (21.3)	9 (28.1)	0.596
Immobilization (%)	7 (14.9)	7 (21.9)	0.552
History of thromboembolic disease (%)	5(10.6)	5 (15.6)	0.540
Active Cancer, *n* (%)	3 (6.4)	6 (18.8)	0.149
**Clinical presentation**			
Sudden Dyspnea, *n* (%)	40 (85.1)	30 (93.75)	1
Syncope, *n* (%)	29 (61.7)	10 (31.3)	0.005
Chest pain, *n* (%)	10 (21.3)	10 (31.3)	0.598
Fever, *n* (%)	0	4 (12.5)	0.030
Hemoptysis, *n* (%)	0	1 (3.1)	0.427
Lung infarction, *n* (%)	17 (36.2)	7 (21.9)	0.210
Inferior Vena Cava Reflux, *n* (%)	21 (44.7)	17 (53.1)	0.797
**Laboratory at admission**			
Lactate [mg/dl], median [IQR]	74.9 [40.75–127.8]	12.7 [8.2–22.7]	<0.001
pH, median [IQR]	7.238 [6.99–7.33]	7.436 [7.36–7.47]	<0.001
PaO_2_ [mmHg], median [IQR]	113 [72.1–203.9]	69.3 [51.6–88.9]	<0.001
PaCO_2_ [mmHg], median [IQR]	54.2 [42.4–74.8]	35.6 [29.8–41]	<0.001
Creatinine [mg/dl], mean (SD)	1.54 [1.19–2.2]	0.94 [0.66–1.26]	<0.001
Bilirubin [mg/dl], median [IQR]	1 [0.5–1.6]	0.8 [0.5–1]	0.097
AST [U/I], median [IQR]	244.5 [110.3–681]	28 [17.3–54.5]	<0.001
ALT [U/I], median [IQR]	242.5 [80.3–440.3]	27.5 [13–80.3]	<0.001
GGT [U/I], median [IQR]	95 [42–191]	39 [22.8–67.3]	0.002
Hemoglobin [g/dl], median [IQR]	12.9 [11.2–14.4]	11.8 [10.4–13.3]	0.148
Leukocyte count [G/l], median [IQR]	15 [10.4–20.6]	12.7 [7.9–14.9]	0.016
Platelet count [G/l], median [IQR]	176 [123–230.8]	211 [174–290]	0.07
Fibrinogen [mg/dl], median [IQR]	1.8 [0.5–3.5]	4.5 [3.4–5.5]	0.003
aPTT [sec], median [IQR]	46 [30.8–88.3]	32.1 [25.2–36.9]	0.001
INR, median [IQR]	1.58 [1.33 2.5]	1.23 [1.12–1.32]	<0.001
HS Troponin I	1727.7 [3.7–3960]	44.2 [1.03–392.4]	0.011
C-reactive protein [mg/dl], median [IQR]	3.91 [1.4–10.5]	5.27 [2.7–16.78]	0.080
**Severity scoring systems at admission**			
SAPS II score, median [IQR]	76 [60–90]	30 [22–35.8]	<0.001
SOFA score, median [IQR]	11 [8–13]	5 [3–6]	<0.001
**Supportive care**			
Mechanical Ventilation, *n* (%)	38 (80.9)	7 (21.9)	<0.001
Noradrenaline, *n* (%)	42 (89.4)	17 (53.1)	<0.001
Dobutamine, *n* (%)	31 (66)	9 (28.1)	0.001
Adrenaline, *n* (%)	22 (46.8)	1 (3.1)	<0.001
Inferior Vena Cava Filter, *n* (%)	9 (19.1)	13 (40.6)	0.122
VA-ECMO, *n* (%)	9 (19.1)	0	0.009
**Treatment**			
Un-fractioned Heparin, *n* (%)	36 (76.6)	26 (81.3)	1
Systemic thrombolysis, *n* (%)	39 (83)	14 (43.8)	0.001
Alteplase, *n* (%)	31 (66)	10 (31.3)	0.006
Tenecteplase, *n* (%)	7 (14.9)	2 (6.3)	0.300
Catheter directed therapies, *n* (%)	16 (34)	12 (37.5)	0.803
Surgical embolectomy, *n* (%)	1 (2.1)	0	1
**Treatment sequence**			
Anticoagulation alone, *n* (%)	3 (6.4)	5 (15.6)	0.001
First line Systemic thrombolysis, *n* (%)	38 (80.9)	13 (40.6)	
First line Catheter directed Therapies, *n* (%)	5 (10.6)	14 (43.8)	
First line Surgical embolectomy, n (%)	1 (2.1)	0	
**Vital signs**			
Systolic blood pressure (mmHg), median [IQR]	90 [81.3–107]	110 [91.5–126]	0.006
Mean blood pressure (mmHg), median [IQR]	65 [59.5–76]	76 [65–85]	0.012
Heart rate (bpm), median [IQR]	102 [87.8–120]	87 [85–110]	0.029
SpO_2_/FiO_2_ ratio, ± SD	203 ± 83	260 ± 100	0.013
Cardiac arrest at presentation, *n* (%)	40 (95)	0	<0.001
**Vital signs at 48 h**			
Systolic blood pressure (mmHg), median [IQR]	104 [90–123]	120 [111–125]	0.014
Mean blood pressure (mmHg), median [IQR]	69 [65–81]	78 [75–93]	0.002
Heart rate (bpm), median [IQR]	100 [86–110]	87 [82–97]	0.015
SpO_2_/FiO_2_ ratio, ± SD	283 ± 119	344 ± 93	0.036
**Outcomes**			
Major bleeding *, *n* (%)	14 (29.8)	6 (18.8)	0.194
Stroke, n (%)	4 (8.5)	0	0.129
Recurrent PE, *n* (%)	1 (2.1)	4 (12.5)	0.159
Ventilator Associated Pneumonia, *n* (%)	6 (12.8)	5 (15.6)	1
Multiorgan failure, *n* (%)	16 (34)	2 (6.3)	0.002
Sepsis, *n* (%)	5 (10.6)	4 (12.5)	1
Acute Kidney Injury, *n* (%)	27 (57.4)	12 (37.5)	0.037
Renal replacement therapy, *n* (%)	5 (10.6)	1 (3.1)	0.231
ICU LOS, days, median [IQR]	6 [1–19.5]	5 [4–22]	0.302
LOS, days, median [IQR]	7 [1–36]	15 [8.5–29.3]	0.05
7-day mortality, *n* (%)	19 (40.4)	3 (9.4)	0.002
30-day mortality, *n* (%)	23 (48.9)	4 (12.5)	0.001
90-day mortality, *n* (%)	23 (48.9)	6 (18.8)	0.009
Reperfusion failure, *n* (%)	16 (34)	3 (9.4)	0.015

COPD: Chronic Obstructive Pulmonary Disease; CTPA: computed tomography pulmonary angiography; ICU: Intensive Care Unit; LOS: length of stay; TTE: transthoracic echocardiography; VA-ECMO: Venoarterial extracorporeal membrane oxygenation. * According to control of Anticoagulation Subcommittee of the ISTH.

## Data Availability

The data that support the findings of this study are available from the corresponding author upon reasonable request.

## Supplementary Materials

The following supporting information can be downloaded at: https://www.mdpi.com/article/10.3390/jcdd12120459/s1, Table S1: STROBE checklist.

## Abbreviations

The following abbreviations are used in this manuscript:

ALT	Alanin Aminotransferase
AST	Aspartate Aminotransferase
COPD	Chronic Obstructive Pulmonary Disease
CTPA	Computed Tomography Pulmonary Angiography
CPR	Cardiopulmonary Resuscitation
ICU	Intensive Care Unit
LOS	Lenght of Stay
PE	Pulmonary Embolism
ROSC	Return of Spontaneous Circulation
TTE	Transthoracic Echocardiography
VA-ECMO	Venoarterial Extracorporeal Membrane Oxygenation
eCPR	Venoarterial Extracorporeal Membrane Oxygenation during CPR

## Appendix A

**Table A1:** Baseline Characteristics of 79 patients with High-Risk PE.

Demographics	
Age (years) ± SD	61.8 ± 15.3
Sex (female) *n* (%)	43 (54.4%)
Body Mass Index (kg/m^2^) ± SD	29.9 ± 7.7
**Past medical history**	
Smoking *n* (%)	32 (40.5)
Arterial hypertension, *n* (%)	33 (41.8)
Dyslipidemia, *n* (%)	29 (36.7)
History of cancer, *n* (%)	16 (20.3)
Diabetes mellitus, *n* (%)	11 (13.9)
Chronic obstructive pulmonary disease, *n* (%)	9 (11.4)
Chronic renal disease, *n* (%)	6 (7.6)
History of stroke, *n* (%)	5 (6.3)
History of liver disease, *n* (%)	3 (3.8)
Atrial fibrillation, *n* (%)	3 (3.8)
Coronary artery disease, *n* (%)	2 (2.5)
Previous myocardial infarction, *n* (%)	2 (2.5)
Previous percutaneous coronary intervention, *n* (%)	2 (2.5)
History of coagulopathy, *n* (%)	1 (1.3)
History of platelet dysfunction, *n* (%)	1 (1.3)
Chronic heart failure, *n* (%)	1 (1.3)
**Predisposing factors for venous thromboembolism**	
Prolonged bed rest, *n* (%)	19 (24.1)
Surgery 1–2 weeks previous (%)	13 (16.5)
Immobilization, *n* (%)	14 (17.7)
History of thromboembolic disease, *n* (%)	10 (12.7)
Active Cancer, *n* (%)	9 (11.4)
Deep vein thrombosis, *n* (%)	8 (10.1)
Hormonal contraceptives, *n* (%)	4 (5.1)
**Clinical Presentation**	
Sudden Dyspnea, *n* (%)	70 (88.6)
Cardiac arrest, *n* (%)	40 (50.6)
Syncope, *n* (%)	39 (49.4)
Cardiogenic shock, *n* (%)	31 (39.2)
Chest pain, *n* (%)	20 (25.3)
Fever, *n* (%)	4 (5.1)
Hemoptysis, *n* (%)	1 (1.3)
Cardiopulmonary resucitation, *n* (%)	40 (50.6)
CPR duration (min), median [IQR]	24 [9–43]
eCPR, *n* (%)	3 (3.8)
**Diagnostic tests**	
CTPA, *n* (%)	76 (96.2)
TTE at presentation, *n* (%)	26 (32.9)
RV dysfunction, *n* (%); n = 26	26 (100)
TAPSE [mm], (SD); *n* = 17	15.6 ± 9.6
PASP [mmHg], (SD); *n* = 12	38 ± 29.9
Intracavitary thrombus (%); *n* = 17	1 (5.6)
**Thrombophilia Screening**	
ATIII level (%), median [IQR] (*n* = 29)	108 [96–120]
Protein C level (%), median [IQR] (*n* = 30)	110 [88.5–139]
Protein S level (%), median [IQR] (*n* = 29)	90 [80–98]
AntiXa (U/mL), median [IQR] (*n* = 19)	0.64 [0.43–1.07]
Factor Leiden heterozigous mutation, *n* (%) (*n* = 31)	2 (6.5)
Prothrombin gene heterozygous mutation, *n* (%) (*n* = 31)	2 (6.5)
**Treatment**	
Un-fractioned Heparin, *n* (%)	62 (78.5)
Argatroban, *n* (%)	1(1.3)
Lepirudin, *n* (%)	1(1.3)
Systemic thrombolysis, *n* (%)	53 (67%)
Pre-hospital ST, *n* (%); *n* = 53	15 (28.3)
In-hospital ST, *n* (%); *n* = 53	38 (71.7)
Catheter directed therapies, *n* (%)	28 (35.4)
Endovascular thrombectomy, *n* (%); *n* = 28	25 (89.3)
Catheter directed thrombolysis, *n* (%); *n* = 28	6(21.4)
Surgical embolectomy, *n* (%)	1(1.3)
**Support treatment**	
Mechanical Ventilation, *n* (%)	45 (58.4)
Norepinephrine, *n* (%)	59 (74.7)
Epinephrine, *n* (%)	23 (29.1)
Dobutamine, *n* (%)	40 (50.6)
Vasopressin *n* (%)	1 (1.3)
VA-ECMO *n* (%)	9 (11.4)
**Vital signs**	
Systolic blood pressure [mmHg], median [IQR]	115 [101–125]
Mean blood pressure [mmHg], median [IQR]	77 [70–87]
Heart rate [beats per minute], median [IQR]	90 [84–105]
SpO_2_/FiO_2_ ratio, ± SD	316 ± 108
**Outcomes**	
Major bleeding *, *n* (%)	20 (25.3)
Reperfusion failure, *n* (%)	19 (24)
Stroke, *n* (%)	4 (5.1)
Recurrent PE, *n* (%)	5 (7.1)
Ventilator Associated Pneumonia, *n* (%)	11 (13.9)
Multiorgan failure, *n* (%)	18 (22.8)
Sepsis, *n* (%)	9 (11.4)
Acute Kidney Injury, *n* (%)	39 (49.4)
Renal replacement therapy, *n* (%)	6 (7.6)
ICU LOS, days, median [IQR]	5 [2–20]
LOS, days, median [IQR]	13 [4–32]
7-day mortality, *n* (%)	22 (27.8)
30-day mortality, *n* (%)	27 (34.2)
90-day mortality, *n* (%)	29 (36.7)

ATIII: antithrombin III level; CTPA: computed tomography pulmonary angiography; ICU: Intensive Care Unit; LOS: length of stay; TTE: transthoracic echocardiography; eCPR: Venoarterial extracorporeal membrane oxygenation during cardiopulmonary resuscitation. * According to control of Anticoagulation Subcommittee of the ISTH.
