# Supplementary material for: Clinical Outcomes and Treatment Strategies in Catastrophic High-Risk Pulmonary Embolism: A Retrospective Analysis
Source: J Cardiovasc Dev Dis. 2025 Nov 25;12(12):459. doi: 10.3390/jcdd12120459 (PMC12734340; doi:10.3390/jcdd12120459)
Supplement: Supplementary file 1 [file jcdd-12-00459-s001.zip › jcdd-3954717-supplementary.pdf]

# STROBE Checklist v4.0 - Completed for Manuscript: Clinical outcomes and treatment strategies in Catastrophic High-risk Pulmonary Embolism

| STROBE Item | Manuscript Reference |
| --- | --- |
| Title and Abstract | The title clearly identifies the study as a retrospective analysis. The abstract summarizes objectives, methods, results, and conclusions. |
| Background/Rationale | The introduction provides background on high-risk PE and the rationale for distinguishing catastrophic PE. |
| Objectives | The study aims to compare clinical characteristics, management strategies, and outcomes between catastrophic and non-catastrophic high-risk PE. |
| Study Design | A retrospective cohort design is described in the methods section. |
| Setting | The study was conducted at a single tertiary center from 2018 to 2024. |
| Participants | Inclusion criteria are patients diagnosed with high-risk PE. Catastrophic PE is defined using specific clinical criteria. |
| Variables | Variables include clinical characteristics, treatments, and outcomes. Definitions for catastrophic PE and major bleeding are provided. |
| Data Sources/Measurement | Data were collected from hospital records. Imaging and laboratory tests are described. |
| Bias | Potential bias due to single-center design and retrospective nature is acknowledged in the limitations. |
| Study Size | The study includes 79 patients. |
| Quantitative Variables | Continuous variables are analyzed using Mann-Whitney U test. Categorical variables use chi-squared or Fisher's exact tests. |
| Statistical Methods | Kaplan-Meier survival analysis and p-values are reported. Statistical significance is defined as $p<0.05$. |
| Participants (Results) | Flow and characteristics of participants are described in the results section. |
| Descriptive Data | Baseline characteristics are presented in Table 1 and Supplementary Table 1. |
| Outcome Data | Mortality rates and complications are reported for both groups. |

| | |
|---|---|
| Main Results | Differences in clinical presentation, treatment, and outcomes between groups are statistically analyzed. |
| Other Analyses | Subgroup comparisons between catastrophic and non-catastrophic PE are included. |
| Key Results | Catastrophic PE is associated with worse outcomes and higher treatment intensity. |
| Limitations | Limitations include retrospective design, single-center setting, and small sample size. |
| Interpretation | Findings are interpreted in the context of existing literature and clinical implications. |
| Generalisability | Generalizability is limited due to single-center design. |
| Funding | No external funding was received. |
| Ethics Approval | Approved by the Institutional Review Board of Hospital Clínic de Barcelona (HCB/2025/0169). |
